# Adjacent segment degeneration may predict significantly worse leg pain outcomes after lumbar discectomy

**DOI:** 10.1007/s00256-026-05130-5

**Published:** 2026-01-22

**Authors:** Tero Korhonen, Jyri Järvinen, Juha Pesälä, Marianne Haapea, Juhani Määttä, Jaakko Niinimäki, Pietari Kinnunen

**Affiliations:** 1 Department of Diagnostic Radiology, Oulu University Hospital, Oulu, PO Box 10, 90029 OYS Finland; 2 Department of Orthopedics and Traumatology, Oulu University Hospital, Oulu, Finland; 3 Medical Research Center Oulu, Oulu University Hospital and University of Oulu, Oulu, Finland; 4 Research Service Unit, Oulu University Hospital, Oulu, Finland; 5 Research Unit of Health Sciences and Technology, University of Oulu, Oulu, Finland

**Keywords:** Lumbar, Discectomy, Outcome, Modic changes, Endplate damage, Intervertebral disc degeneration

## Abstract

**Objective:**

We assessed whether preoperative advanced multisegmental degeneration is associated with worse 1-year outcomes of primary single-level lumbar discectomy.

**Materials and methods:**

A literature-based scoring system was developed to quantify degeneration in the operated and adjacent lumbar segments, based on advanced phenotypes of common intervertebral disc–related degenerative features. Each segment received a score from 0 to 3: cranial and caudal ≥ 25% endplate damage (EPD) and Modic changes type I (MC1) were assigned 0.5 points each, and Pfirrmann grade ≥ 4 was assigned 1 point. The final adjacent segment degeneration score was calculated as the mean of the operated and adjacent segment scores. Prospectively collected data from primary single-level lumbar discectomy patients operated in a single center between 2017 and 2022 were retrospectively analyzed. Patients were stratified into none-to-mild (≤ 0.5), moderate (0.5 < score < 1.33), and severe (≥ 1.33) degeneration groups, using the 40th and 80th percentile cut-offs. A linear mixed-effects model was employed to assess between-group differences in 1-year improvements in low back (LBP) and leg pain (VAS, 0–100), disability (ODI), and quality of life (EQ-5D-3L).

**Results:**

Among the 140 patients included (mean age 45.3 years; 57.9% male), all PROMs improved overall. The severe group showed significantly smaller improvements from baseline to 1-year follow-up, with adjusted mean differences in change of 20.2 for LBP, 31.6 for leg pain, and 11.1 for disability relative to the none-to-mild reference group.

**Conclusion:**

Severe preoperative adjacent segment degeneration may be associated with smaller 1-year improvements in pain and disability after primary single-level lumbar discectomy.

**Supplementary Information:**

The online version contains supplementary material available at 10.1007/s00256-026-05130-5.

## Introduction

The recurrence of low back pain (LBP) and leg pain following lumbar discectomy remains a significant clinical challenge. In a retrospective register study of patients undergoing discectomy for lumbar disc herniation (LDH), 36% and 29% of patients failed to achieve the minimal clinically important difference (MCID) for LBP at 1 and 2 years postoperatively, respectively [1]. Similarly, review studies indicate that clinically significant postoperative LBP or leg pain occurs in 3–34% and 5–36% of patients in the short and long term, respectively [2, 3]. The postoperative progression of segmental degeneration at the operated level may contribute to such pain [4–7]. Consistent with this, several studies have associated certain preoperative degenerative features of the operated segment with poorer discectomy outcomes [8–10].

Meanwhile, the burden of degenerative features in the lumbar spine has been quantified both at the level of the entire lumbar spine, using sum scores, and at the level of individual lumbar segments, by evaluating the co-occurrence of the features. Notably, both approaches have demonstrated clinical significance, as a greater number of degenerative features has been correlated with higher levels of LBP [11–15]. To date, no previous research using these methods has assessed the influence of preoperative adjacent segment degeneration on lumbar discectomy outcomes.

Thus, this study aimed to assess the impact of preoperative advanced adjacent segment degeneration on 1-year outcomes of primary single-level lumbar discectomy. The methodologies from previous studies of calculating a sum score of lumbar spine degenerative features and assessing their co-occurrence were combined into a novel literature-based scoring system.

## Materials and methods

### Study population

The study population, extracted from the Finnish spine register (FinSpine) [16], included consecutive patients who underwent primary single-level lumbar discectomy for LDH at a tertiary-level hospital between October 2017 and September 2022. Patients were excluded if they had a history of prior lumbar surgery, if the interval between preoperative MRI and surgery exceeded 6 months, if data on preoperative or 1-year postoperative patient-reported outcome measures (PROMs) were missing, or if they underwent reoperation during the follow-up period.

Prospectively collected demographic and clinical data, including age, sex, body mass index (BMI), smoking status, symptom duration, preoperative mental health status, surgical level, preoperative cauda equina syndrome, and preoperative motor deficit of leg impairing walking (leg strength ≤ 3 out of 5), were extracted from the register.

The extracted prospectively collected preoperative and 1-year postoperative PROM data comprised LBP and leg pain measured on a Visual Analogue Scale (VAS, 0–100), disability assessed using the Oswestry Disability Index v. 2.0 (ODI, 0–100) [17], and health-related quality of life (QoL) measured with the EQ-5D-3L scale [18]. The EQ-5D-3L comprises two components: a descriptive system assessing various aspects of QoL, which were converted into index values (EQ-index, − 0.011 to 1.000, where 1.000 represents the optimal health state) using the Finnish valuation set from the EuroQol office; and a VAS for overall health (EQ-VAS, 0–100), where higher scores indicate better QoL. Notably, the EQ-VAS slider was oriented horizontally in the register software, unlike the EuroQoL-recommended vertical layout.

### MRI and image analysis

All patients underwent preoperative 1.5 T or 3 T lumbar MRI, including T1- and T2-weighted sagittal, T2-weighted axial, and either coronal or sagittal fat-saturated T2-weighted sequences. The MRIs were analyzed on clinical workstations by two senior musculoskeletal radiologists with 20 and 14 years of experience in musculoskeletal imaging, respectively (JN, who reviewed 89/140 [63.6%] of the MRIs, and JJ, who reviewed 51/140 [36.4%]). The radiologists were blinded to all demographic and clinical data except for the operated lumbar segment. The operated and adjacent lumbar segments were evaluated for endplate damage (EPD), Modic changes (MC), and intervertebral disc (IVD) degeneration, as detailed below. For intra- and interobserver reliability assessments, both radiologists re-evaluated 15 MRIs they had previously reviewed, and an additional 19 randomly selected MRIs were independently analyzed by both radiologists, without access to their prior or each other’s findings. To increase sample size in reliability analysis, all lumbar segments were included. This resulted in the re-assessment of 75 IVD degeneration ratings and 150 EPD and MC ratings for intra-observer reliability and 95 IVD degeneration ratings and 190 EPD and MC ratings for inter-observer reliability.

### Scoring system for adjacent segment degeneration

To quantify the presence of preoperative degenerative features in the operated and adjacent lumbar segments, a scoring system was developed. This system categorized the included degenerative features—EPD, MC, and IVD degeneration—into advanced and non-advanced phenotypes (Table 1). Literature-based rationales for the cut-off values of advanced phenotypes are provided in the Supplemental Material. EPD and MC were evaluated separately for the cranial and caudal regions of each lumbar segment, with their advanced phenotypes assigned 0.5 points each. Advanced IVD degeneration was allocated one point. Consequently, the score for each lumbar segment ranged from 0 to 3 points.

**Table 1:** The scoring scheme for the individual lumbar segments

Feature	Criterion for advanced phenotype	Points for each advanced phenotype	Maximum points for each segment
Endplate damage (EPD)	Area of damage ≥ 25% relative to the affected endplate	0.5	1
Modic changes (MC)	Pure or predominant Modic changes type I [34]	0.5	1
Intervertebral disc degeneration	Pfirrmann grade ≥ 4 [35]	1	1

The scores of each lumbar segment were used to calculate the final score for adjacent segment degeneration. For discectomies performed on segments other than L1/L2 or L5/S1, the score was calculated as the mean value of the individual scores from the cranial adjacent segment, the operated segment, and the caudal adjacent segment. For surgeries at L1/L2 or L5/S1, where only one adjacent lumbar segment is present, the score was derived as the mean of the individual scores from the operated segment and the single adjacent lumbar segment. Consequently, the final score representing the degree of adjacent segment degeneration ranged from 0 to 3 (Table 2).

**Table 2:** The scoring scheme for the final adjacent segment degeneration score

Operated segment	Scoring method	Range (min–max)	Grading
L2/L3, L3/L4, or L4/L5	Mean value of the scores from the cranial adjacent segment, the operated segment, and the caudal adjacent segment	0–3	None-to-mild: ≤ 0.5 Moderate: 0.5 < score < 1.33 Severe: ≥ 1.33
L1/L2 or L5/S1	Mean value of the scores from the operated segment and the single adjacent lumbar segment	0–3	

### Statistical analysis

Patients were categorized into three groups of adjacent segment degeneration: none-to-mild, moderate, and severe. Given that lumbar degenerative changes are notably prevalent in asymptomatic patients within the age range of the present study [19, 20], our aim was to identify a potentially clinically significant subgroup at the extreme end of the degeneration spectrum. Therefore, the cut-off for the severe group was set at the 80th percentile of the score range, including patients with scores of ≥ 1.33 (24/140 [17.1%] patients). The 40th percentile was used to distinguish between the none-to-mild and moderate groups, with scores of ≤ 0.5 assigned to the none-to-mild group (59/140 [42.1%] patients) and scores where 0.5 < score < 1.33 to the moderate group (57/140 [40.7%] patients) (Table 2).

Descriptive data were presented as means ± standard deviations (SD), medians with interquartile ranges (IQR), or frequencies with percentages. Continuous variables were analyzed using one-way analysis of variance (ANOVA) and Kruskal–Wallis tests (for normally and non-normally distributed data, respectively), while dichotomous variables were evaluated with chi-square tests. Post hoc comparisons were conducted using Tukey’s test and Holm-Bonferroni-corrected pairwise analyses. To ensure statistical feasibility, certain subgroups of demographic data were combined: symptom duration was categorized as < 12 weeks, 3–12 months, or > 12 months, and preoperative anxiety or depression was classified as present (EQ-5D-3L Question 5: scores 2–3) or absent (score 1). Due to its exceedingly low occurrence (3/140 [2.1%]), preoperative cauda equina syndrome was excluded from the analysis.

The effect of the adjacent segment degeneration grades on 1-year lumbar discectomy outcomes was analyzed using linear mixed-effects models. The PROM parameter of interest was used as the dependent variable, with the patient serving as the subject factor. The primary estimand was the change in the PROM parameter between baseline and 1 year, and the adjusted difference in this change between groups (represented by the regression coefficient for the interaction term) served as the summary metric. To evaluate whether the effect of surgery on outcomes differed between the groups, the model included an interaction term “follow-up * group” across the baseline and follow-up timepoints. Adjustments were made for clinically important covariates: age, sex, BMI, smoking status, symptom duration, preoperative mental health status, and preoperative motor deficit of leg. Further information on the adjusted covariates is provided in the Supplemental Material. Surgical level, and whether the score was based on two or three levels, was not adjusted for. Baseline differences were assessed using ANOVA or chi-square tests and the mixed-effects model adjusted for baseline PROM values. Results are presented as PROM estimates and regression coefficients with standard errors (s.e.); 95% confidence intervals (CIs) can be calculated using the formula: estimate ± 1.96 * s.e.

Inter- and intraobserver reliabilities were assessed by calculating Cohen’s kappa (*κ*) and prevalence-adjusted bias-adjusted kappa (PABAK) [21] for detecting the advanced phenotypes included in the scoring system throughout the lumbar spine. The unit of analysis was segment for Pfirrmann and endplate for EPD and MC. Unweighted *κ* was used for ordinal ratings, and 95% CIs for *κ* and PABAK were computed using the asymptotic method. The *κ* and PABAK values were interpreted according to Landis and Koch [22].

A *p*-value < 0.05 was considered statistically significant throughout the statistical tests. Statistical analyses were performed using IBM SPSS, v. 29 (IBM Corp., Armonk, NY, USA) except for the reader reliability testing, which was done using R v. 4.3.0 (R Foundation for Statistical Computing, Vienna, Austria) with the “EpiR” and “vcs” packages.

## Results

### Demographic and clinical characteristics

**Fig. 1:**
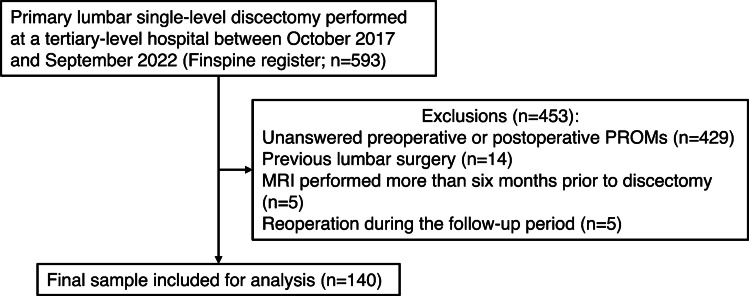
A flow chart of the study showing the exclusion rates and reasons

Of the 593 lumbar discectomy patients identified in the register, 453 (76.4%) were excluded due to missing preoperative or postoperative PROMs (*n* = 429), previous lumbar surgery (*n* = 14), MRI performed more than six months before discectomy (*n* = 5), or reoperation during the follow-up period (*n* = 5), leaving 140 patients (23.6%) for analysis (mean age: 45.3 ± 14.0 years; 81 [57.9%] male). Microdiscectomy was performed for 136 (97.1%) patients, while four (2.9%) underwent endoscopic discectomy. Figure 1 presents a flow chart of the study including the exclusion rates and reasons, while Table 3 summarizes the patients’ demographic and clinical characteristics by group.

**Table 3:** Demographic and clinical baseline characteristics of the total study sample and by group

	Total	None-to-mild	Moderate	Severe	*p*^a^
*N* (%)	140 (100.0%)	59 (42.1%)	57 (40.7%)	24 (17.1%)	
Age, mean (SD)	45.3 (14.0)	39.7 (12.0)	46.4 (13.3)	56.9 (12.9)	< 0.001
Sex					0.787
Male, *N* (%)	81 (57.9%)	36 (61.0%)	31 (54.4%)	14 (58.3%)	
Female, *N* (%)	59 (42.1%)	23 (39.0%)	26 (45.6%)	10 (41.7%)	
BMI, mean (SD)	28.0 (5.5)	28.3 (6.3)	28.1 (5.1)	27.3 (4.1)	0.743
Active smoking					0.608
Yes, *N* (%)	27 (19.3%)	11 (18.6%)	13 (22.8%)	3 (12.5%)	
No, *N* (%)	110 (78.6%)	46 (78.0%)	44 (77.2%)	20 (83.3%)	
Missing data, *N* (%)	3 (2.1%)	2 (3.4%)	-	1 (4.2%)	
Preoperative anxiety					0.746
Yes, *N* (%)	39 (27.9%)	15 (25.4%)	16 (28.1%)	8 (33.3%)	
No, *N* (%)	99 (70.7%)	43 (72.9%)	41 (71.9%)	15 (62.5%)	
Missing data, *N* (%)	2 (1.4%)	1 (1.7%)	-	1 (4.2%)	
Duration of symptoms					0.035
< 6 weeks, *N* (%)	22 (15.7%)	7 (11.9%)	10 (17.5%)	5 (20.8%)	
6–12 weeks, *N* (%)	28 (20.0%)	15 (25.4%)	9 (15.8%)	4 (16.7%)	
3–12 months, *N* (%)	58 (41.4%)	30 (50.8%)	23 (40.4%)	5 (20.8%)	
> 12 months, *N* (%)	32 (22.9%)	7 (11.9%)	15 (26.3%)	10 (41.7%)	
Level of herniation					0.144
L1–L2, *N* (%)	-	-	-	-	
L2–L3, *N* (%)	5 (3.6%)	1 (1.7%)	3 (5.3%)	1 (4.2%)	
L3–L4, *N* (%)	14 (10.0%)	4 (6.8%)	5 (8.8%)	5 (20.8%)	
L4–L5, *N* (%)	66 (47.1%)	26 (44.1%)	26 (45.6%)	14 (58.3%)	
L5–S1, *N* (%)	55 (39.3%)	28 (47.5%)	23 (40.4%)	4 (16.7%)	
Preoperative motor deficit of leg impairing walking					0.634
Yes, *N* (%)	44 (31.4%)	16 (27.1%)	20 (35.1%)	8 (33.3%)	
No, *N* (%)	96 (68.6%)	43 (72.9%)	37 (64.9%)	16 (66.7%)	
Preoperative cauda equina syndrome					0.820
Yes, *N* (%)	3 (2.1%)	2 (3.4%)	1 (1.8%)	0 (0%)	
No, *N* (%)	137 (97.9%)	57 (96.6%)	56 (98.2%)	24 (100.0%)	
Preoperative PROMs assessment-operation interval, median (IQR), days	6.0 (2.3–12.0)	5.0 (2.0–12.0)	6.0 (2.0–12.5)	6.0 (3.0–13.5)	0.985
MRI-operation interval, median (IQR), days	29 (9–64)	30 (8–64)	28 (10–66)	27 (9–66)	0.900

^a^Continuous variables were compared using one-way ANOVA or Kruskal–Wallis tests (for normally and non-normally distributed variables), and categorical variables using chi-square tests

### Degenerative characteristics

A total of 59 (42.1%) patients were classified into the none-to-mild group, 57 (40.7%) into the moderate group, and 24 (17.1%) into the severe group of adjacent segment degeneration. Mean degeneration scores of each group are presented in Table 4, and frequency tables of the different degeneration scores and phenotypes are provided in the Supplemental Material.

**Table 4:** Overview of adjacent segment degeneration scores for the total study sample and by group

	Total	None-to-mild	Moderate	Severe
Adjacent segment degeneration score, mean (SD)	0.75 (0.48)	0.30 (0.22)	0.90 (0.19)	1.48 (0.21)

Intra- and interobserver reliabilities were substantial to almost perfect for detecting the included advanced phenotypes (*κ* = 0.61–1.00; PABAK = 0.88–1.00 for intraobserver reliability; *κ* = 0.69–0.91; PABAK = 0.88–0.92 for interobserver reliability), with the exception of the moderate *κ* value for interobserver reliability in detecting the MC phenotype (*κ* = 0.42; PABAK = 0.89). Exact *κ* and PABAK values with 95% CIs are provided in the Supplemental Material.

### Discectomy outcome analysis

In the fully adjusted model, controlling for age, sex, BMI, smoking status, symptom duration, preoperative mental health status, and preoperative motor deficit of leg, all groups showed improvement in all PROMs following discectomy. Meanwhile, significant between-group differences were noted in the improvement rates of leg pain (*p* = 0.008, Table 5).

**Table 5:** Estimated means of the groups’ patient-reported outcome measures (PROMs)

	None-to-mild	Moderate	Severe
LBP, est. mean (s.e.)			
Baseline	57.9 (5.3)	54.3 (4.8)	49.9 (7.3)
Follow-up	28.2 (5.4)	27.0 (4.8)	40.4 (7.3)
*p*^a^	0.072		
Leg pain, est. mean (s.e.)			
Baseline	70.4 (4.8)	74.1 (4.6)	55.6 (6.6)
Follow-up	25.9 (5.6)	30.5 (5.1)	42.7 (7.8)
*p*^a^	0.008		
Disability, est. mean (s.e.)			
Baseline	48.5 (2.6)	47.5 (2.3)	41.2 (3.7)
Follow-up	17.0 (3.1)	17.1 (2.8)	20.8 (4.3)
*p*^a^	0.095		
EQ-index, est. mean (s.e.)			
Baseline	0.45 (0.020)	0.45 (0.018)	0.46 (0.028)
Follow-up	0.68 (0.035)	0.68 (0.032)	0.63 (0.051)
*p*^a^	0.547		
EQ-VAS, est. mean (s.e.)			
Baseline	41.4 (3.7)	40.9 (3.4)	48.2 (5.3)
Follow-up	68.7 (4.4)	67.2 (4.1)	72.5 (6.3)
*p*^a^	0.936		

^a^For between-group differences in improvement rates

Models were adjusted for clinically important covariates: age, sex, BMI, smoking status, symptom duration, preoperative mental health status, and preoperative motor deficit of leg

95% CIs can be calculated using the formula: estimate ± 1.96 * s.e

**Fig. 2:**
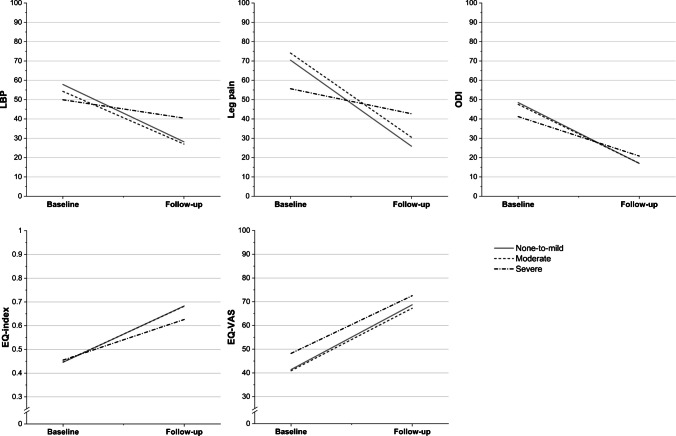
Visual representation of changes in patient-reported outcome measures (PROMs) from baseline to follow-up across the study groups. The estimates and corresponding statistical analyses are provided in Tables 5 and 6

**Fig. 3:**
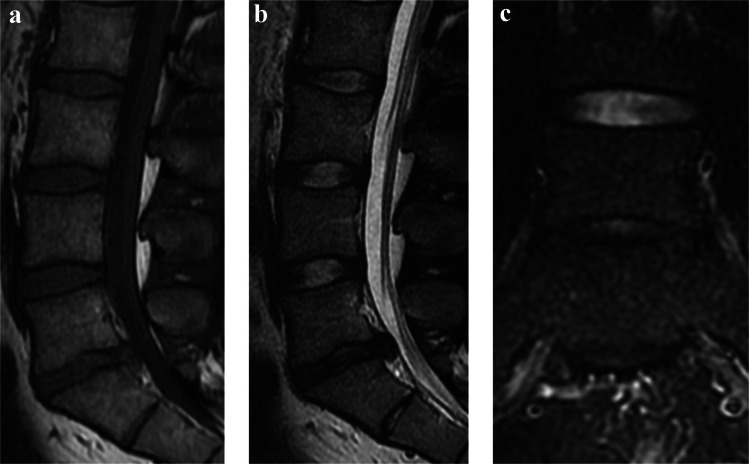
A 23-year-old patient with lumbar disc herniation at L5/S1 shown in preoperative lumbar MRI: sagittal T1-weighted (**a**), sagittal T2-weighted (**b**), and coronal STIR (**c**) sequences. Neither the operated segment nor the adjacent segment (L4/L5) exhibited Modic changes. Minor endplate damage (< 25% of the endplate area) was observed in both endplates of L5/S1. The Pfirrmann grade was 4 at L5/S1, representing the only advanced phenotype in the scoring system (1 point), while the adjacent segment demonstrated a Pfirrmann grade 2 intervertebral disc. With an adjacent segment degeneration score of 0.5, the patient was classified into the none-to-mild degeneration group. The patient underwent discectomy with satisfactory one-year outcomes: leg pain decreased from 65 to 18 and low back pain from 64 to 9 (on a 0–100 VAS), while the ODI score improved from 22 to 8

**Fig. 4:**
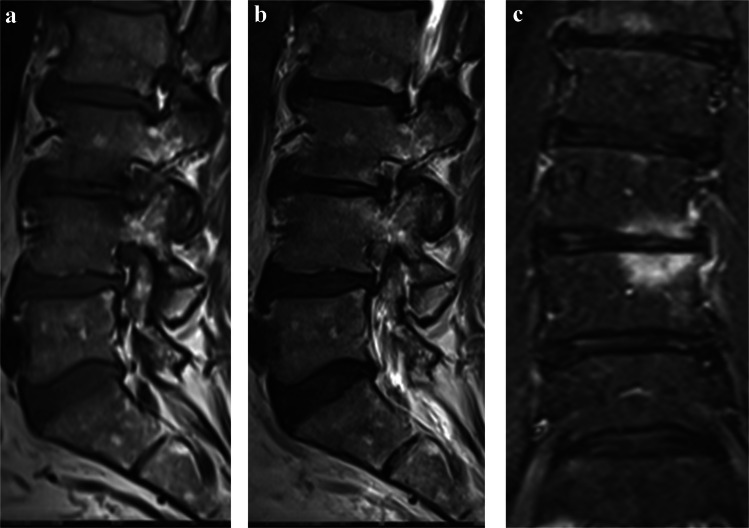
A 64-year-old patient with lumbar disc herniation at L3/L4 shown in preoperative lumbar MRI: sagittal T1-weighted (**a**), sagittal T2-weighted (**b**), and coronal STIR (**c**) sequences. The operated segment exhibited Modic changes: predominant type I (mixed types I and II) in the upper endplate and pure type I in the lower endplate, representing two advanced phenotypes in the scoring system (0.5 points each). The Pfirrmann grade was 4 at L3/L4 and in both adjacent segments (L2/L3 and L4/L5), adding three advanced phenotypes (1 point each). Modic type II changes were present in both endplates of L2/L3, and minor endplate damage (< 25% of the endplate area) was observed throughout the operated and adjacent segments. The total adjacent segment degeneration score was 1.33, classifying the patient into the severe degeneration group. Surgical outcomes at one year were less satisfactory: leg pain decreased from 43 to 35, low back pain changed from 60 to 61, and the ODI score improved from 54 to 24

The regression coefficient (B) for the interaction term “follow-up * severe” in the LBP, leg pain, and disability models was statistically significant: *B* =  + 20.2; s.e. = 9.0; *p* = 0.027 for LBP; *B* =  + 31.6; s.e. = 10.7; *p* = 0.005 for leg pain; and *B* =  + 11.1; s.e. = 5.3;* p* = 0.038 for disability, indicating that the adjusted change in LBP, leg pain, and disability was significantly smaller in the severe group compared to the none-to-mild group across baseline and 1-year postoperative time points (Tables 5 and 6, Fig. 2). Representative patient cases are provided in Figs. 3 and 4.

**Table 6:** Regression coefficients for patient-reported outcome measures (PROMs) by group

	Follow-up			Follow-up * moderate^a^			Follow-up * severe^a^		
	B	s.e	p	B	s.e	p	B	s.e	p
LBP	−29.7	4.9	< 0.001	+ 2.4	6.9	0.723	+ 20.2	9.0	0.027
Leg pain	−44.5	5.9	< 0.001	+ 0.93	8.3	0.911	+ 31.6	10.7	0.005
Disability	−31.5	2.9	< 0.001	+ 1.0	4.0	0.796	+ 11.1	5.3	0.038
EQ-index	+ 0.24	0.035	< 0.001	−0.001	0.049	0.979	−0.066	0.064	0.309
EQ-VAS	+ 27.3	4.4	< 0.001	−0.94	6.2	0.880	−2.9	8.1	0.717

^a^Interaction term “follow-up * group” denotes the adjusted difference in change from baseline to follow-up for each group relative to the none-to-mild reference group

Models were adjusted for clinically important covariates: age, sex, BMI, smoking status, symptom duration, preoperative mental health status, and preoperative motor deficit of leg

95% CIs can be calculated using the formula: estimate ± 1.96 * s.e

## Discussion

In this study, we quantified preoperative adjacent segment degeneration in a lumbar discectomy patient cohort by calculating a mean score of the prevalence of advanced-level phenotypes of common lumbar IVD-related degenerative features in the operated and adjacent segments. Subsequently, the adjacent segment degeneration scores were compared to 1-year discectomy outcomes. Our analysis revealed that severe adjacent segment degeneration was associated with markedly smaller decreases in LBP, leg pain, and disability. Specifically, the severe group had regression coefficients of + 20.2 for LBP, + 31.6 for leg pain, and + 11.1 for disability, indicating that their adjusted improvement across baseline and 1-year follow-up was on average 20.2, 31.6, and 11.1 units less, respectively, compared with the none-to-mild group. The models were adjusted for clinically relevant baseline covariates, including age, sex, BMI, smoking status, symptom duration, preoperative mental health status, and preoperative motor deficit of the leg; age and symptom duration showed significant baseline between-group differences, ensuring that the observed differences were not attributable to these factors. Notably, the observed adjusted differences exceed the MCID of 15 for LBP and leg pain VAS and 10 for disability on the ODI [23].

A potential cause of clinical deterioration following lumbar microdiscectomy is the cascade of changes initiated by the herniation itself and the removal of the herniated IVD. These can result in a reduction of the IVD space, potentially altering the alignment of facet joints and subsequently compressing nearby nerve roots, a phenomenon termed “vertical stenosis” [24]. Bydon et al. reported that 4.0% of patients required decompression surgery at an adjacent lumbar segment, with or without stabilization, within a mean of 3 years following a primary single-level lumbar discectomy [25]. Notably, the only significant risk factor for the surgery was leg pain after the initial operation (OR = 14.23; *p* < 0.001). Thus, in the present study, patients with severe preoperative adjacent segment degeneration may have already experienced increased degenerative stress in the adjacent segments by the 1-year follow-up, potentially presenting as worse LBP, leg pain, and disability outcomes.

The significantly smaller PROM changes observed in the severe group may also be attributed to heightened chemical irritation of the nerve root. Degenerated IVDs are associated with increased secretion of pain-related inflammatory substances [26], and it has also been suggested that sciatic pain caused by LDH may involve a chemical component [27]. A systematic review of 16 studies, despite a considerable risk of bias and partially imprecise outcome measures, found limited evidence that elevated expression of inflammatory substances in serum and IVD biopsies, particularly IL-21, is associated with higher pain levels [28]. Based on their inconclusive findings regarding the link between inflammation and pain intensity, the authors hypothesized that inflammation may play a significant role in a small subgroup of patients with sciatica.

Furthermore, a study by Djuric et al. reported that in patients with preoperative MC2, the highest grade of a three-tier classification of macrophage infiltration into extracted LDH tissue was significantly associated with worse postoperative disability outcomes at 1 year, although no significant difference in leg pain was observed [29]. The authors suggested that this finding was due to macrophages differentiating primarily into pro-inflammatory subtypes in this patient group, thereby contributing to increased disability. Taken together, these findings highlight the potential that the severe group in the present study exhibits a greater postoperative inflammatory pain component, potentially explaining the observed results.

It is well established that radiological segmental degeneration may progress at the operated segment following lumbar discectomy [4, 5, 30, 31]. Additionally, postoperative progression of IVD degeneration in the caudal adjacent segment has been reported [6]. With the progression of segmental degeneration, the resected facet joint at the operated level may become incompetent, leading to axial LBP and disability. Additionally, lumbar discectomy can cause iatrogenic instability in the lumbar spine, resulting in unphysiological loading of the motion segment, which may further contribute to residual symptoms [24, 32, 33]. Thus, the substantially smaller adjusted improvements on LBP and disability observed in the present study may be explained by the severe group’s reduced ability to tolerate the potential biomechanical changes induced by discectomy.

Certain limitations of the present study should be acknowledged. The study was a retrospective analysis of prospectively collected observational data. The exclusion rate was relatively high, primarily due to unanswered PROMs queries. This low response rate likely stems from the novelty of the register in the participating hospital, particularly during the early years of the study [16]. Furthermore, certain demographic and radiological covariates, such as somatic comorbidities, educational level, and facet joint pathology, were not controlled for. Moreover, adjacent segment degeneration scores were averaged across two or three segments depending on surgical level, which introduced variance differences between patients. Given the relatively small sample size, conducting a more granular analysis of the included phenotypes’ effect on the outcomes was not feasible. Instead, we are currently conducting a subsequent national study designed to validate the present findings and examine the components of the criterion in more detail. Additionally, the follow-up period may be insufficient to fully assess the long-term outcomes of the groups.

Nevertheless, the study exhibits certain strengths. The study population likely represents a typical discectomy patient population, and the data were collected prospectively. Furthermore, various PROMs were utilized in the analysis, and the scoring systems were not only grounded in existing literature but also designed to be clinically meaningful.

In conclusion, severe preoperative adjacent segment degeneration, defined by the presence of advanced-level phenotypes of EPD, MC, and IVD degeneration in the operated and adjacent segments, was associated with significantly smaller improvements in LBP, leg pain, and disability at one year following primary single-level lumbar discectomy.

## Supplementary Information

Below is the link to the electronic supplementary material.

ESM 1 (DOCX 67.6 KB)

## Data Availability

The data that support the findings of this study are available from the Finnish Institute for Health and Welfare, but restrictions apply to the availability of these data, which were used under license for the current study and so are not publicly available.

## Abbreviations

LBP: Low back pain
LDH: Lumbar disc herniation
MCID: Minimal clinically important difference
MRI: Magnetic resonance imaging
PROMs: Patient-reported outcome measures
BMI: Body mass index
VAS: Visual Analogue Scale
ODI: Oswestry Disability Index
QoL: Quality of life
EPD: Endplate damage
MC: Modic changes
IVD: Intervertebral disc
SD: Standard deviation
IQR: Interquartile ranges
ANOVA: Analysis of variance
PABAK: Prevalence-adjusted bias-adjusted kappa
OR: Odds ratio
