# Supplementary material for: Adjacent segment degeneration may predict significantly worse leg pain outcomes after lumbar discectomy
Source: Skeletal Radiol. 2026 Jan 22;55(5):1101–10. doi: 10.1007/s00256-026-05130-5 (PMC13018000; doi:10.1007/s00256-026-05130-5)
Supplement: Supplementary file 1 — (DOCX 67.6 KB) [file 256_2026_5130_MOESM1_ESM.docx]

Supplemental material

| Feature | Cut-off value | Rationale |
| --- | --- | --- |
| Endplate damage (EPD) | Area of damage ≥25% relative to the affected endplate | A sum score of approx. 3.5 (range: 2–6) for the standardized width and depth of EPD dimensions (where ≤0.20, 0.21–0.40, and >0.40, corresponding to 1, 2, and 3 points, respectively) is significantly associated with segment-wise advanced IVD degeneration and the presence of MC [1].  An Endplate Score (EPS, 1–6) of ≥4, indicating any EPD more severe than focal disruption, is significantly associated with segment-wise progression of MC and IVD degeneration [2,3]. |
| Modic changes (MC) | Pure or predominant Modic changes type I (MC1) | MC1 is the most biologically active subtype and has been associated with more advanced degeneration of the adjacent IVD [4–6].  Among the subtypes, MC1 exhibits the strongest correlation with LBP [7,8]. |
| Intervertebral disc (IVD) degeneration | Pfirrmann grade ≥ 4 [9] | In grades 4–5, IVD degeneration has progressed to the extent that the IVD space is at least moderately collapsed [9].  Grades 4–5 in the caudal lumbar spine are strongly associated with LBP in middle-aged patient populations [10]. |

Supplemental Table 1. Literature-based rationales for the cut-off values of advanced phenotypes of the included degenerative features.

Coding and reference categories of the covariates adjusted for:

- Age
  - Continuous variable, no handling
- Sex
  - Female (Ref. group)
  - Male
- BMI
  - Continuous variable, no handling
- Smoking status
  - No (Ref. group)
  - Yes
- Symptom duration
  - <12 weeks (Ref. group)
  - 3–12 months
  - >12 months
- Preoperative mental health status
  - Absent anxiety or depression (Ref. group)
  - Present anxiety or depression
- Preoperative motor deficit of leg
  - No (Ref. group)
  - Yes

| Adjacent segment  degeneration score | N (%) |
| --- | --- |
| 0 | 18 (12.9%) |
| 0.167 | 3 (2.1%) |
| 0.333 | 10 (7.1%) |
| 0.500 | 28 (20.0%) |
| 0.667 | 15 (10.7%) |
| 0.750 | 7 (5.0%) |
| 0.833 | 6 (4.3%) |
| 1.000 | 21 (15.0%) |
| 1.167 | 3 (2.1%) |
| 1.250 | 5 (3.6%) |
| 1.333 | 13 (9.3%) |
| 1.500 | 6 (4.3%) |
| 1.667 | 2 (1.4%) |
| 1.833 | 1 (0.7%) |
| 2.000 | 2 (1.4%) |

Supplemental Table 2. A frequency table of the different adjacent segment degeneration scores.

| Phenotypes | N (%) |
| --- | --- |
| None-to-mild group (N=59) |  |
| None | 18 (12.9%) |
| Only Modic changes | 4 (2.9%) |
| Only intervertebral disc degeneration | 32 (22.9%) |
| Endplate damage and intervertebral disc degeneration | 1 (0.7%) |
| Endplate damage and Modic changes | 1 (0.7%) |
| Modic changes and intervertebral disc degeneration | 3 (2.1%) |
| Moderate group (N=57) |  |
| Only intervertebral disc degeneration | 24 (17.1%) |
| Endplate damage and intervertebral disc degeneration | 15 (10.7%) |
| Modic changes and intervertebral disc degeneration | 11 (7.9%) |
| Endplate damage, Modic changes and intervertebral disc degeneration | 7 (5.0%) |
| Severe group (N=24) |  |
| Only intervertebral disc degeneration |  |
| Endplate damage and intervertebral disc degeneration | 7 (5.0%) |
| Modic changes and intervertebral disc degeneration | 4 (2.9%) |
| Endplate damage, Modic changes and intervertebral disc degeneration | 13 (9.3%) |

Supplemental Table 3. Frequency of advanced-level phenotypes by group.

| Feature | Intra (Reader 1) | | | | | |
| --- | --- | --- | --- | --- | --- | --- |
|  | Cohen’s kappa (κ) | | | Prevalence-adjusted bias-adjusted kappa (PABAK) | | |
|  | κ | SE | 95% CI | PABAK | SE | 95% CI |
| EPD | 0.66 | 0.11 | 0.45–0.86 | 0.88 | 0.04 | 0.78–0.94 |
| MC | 0.94 | 0.06 | 0.83–1.0 | 0.99 | 0.01 | 0.93–1.00 |
| IVD degeneration | 1.00 | 0 | 1.00–1.00 | 1.00 | 0 | 0.90–1.00 |

Supplemental Table 4. Exact Cohen’s kappa and prevalence-adjusted bias-adjusted kappa (PABAK) values with 95% CI of the Reader 1’s intraobserver reliability analysis for the detection of the included components of the scoring system.

| Feature | Intra (Reader 2) | | | | | |
| --- | --- | --- | --- | --- | --- | --- |
|  | Cohen’s kappa (κ) | | | Prevalence-adjusted bias-adjusted kappa (PABAK) | | |
|  | κ | SE | 95% CI | PABAK | SE | 95% CI |
| EPD | 0.72 | 0.12 | 0.48–0.95 | 0.93 | 0.03 | 0.85–0.98 |
| MC | 0.61 | 0.13 | 0.35–0.87 | 0.91 | 0.03 | 0.81–0.96 |
| IVD degeneration | 0.94 | 0.04 | 0.86–1.00 | 0.95 | 0.04 | 0.81–0.99 |

Supplemental Table 5. Exact Cohen’s kappa and prevalence-adjusted bias-adjusted kappa (PABAK) values with 95% CI of the Reader 2’s intraobserver reliability analysis for the detection of the included components of the scoring system.

| Feature | Inter (Readers 1 and 2) | | | | | |
| --- | --- | --- | --- | --- | --- | --- |
|  | Cohen’s kappa (κ) | | | Prevalence-adjusted bias-adjusted kappa (PABAK) | | |
|  | κ | SE | 95% CI | PABAK | SE | 95% CI |
| EPD | 0.71 | 0.10 | 0.53–0.90 | 0.92 | 0.03 | 0.83–0.96 |
| MC | 0.42 | 0.15 | 0.12–0.71 | 0.89 | 0.03 | 0.81–0.95 |
| IVD degeneration | 0.91 | 0.04 | 0.82–1.00 | 0.92 | 0.04 | 0.79–0.98 |

Supplemental Table 6. Exact Cohen’s kappa and prevalence-adjusted bias-adjusted kappa (PABAK) values with 95% CI of interobserver reliability analysis for the detection of the included components of the scoring system.

|  | Total | None-to-mild | Moderate | Severe | p^a^ |
| --- | --- | --- | --- | --- | --- |
| LBP, mean (SD) |  |  |  |  |  |
| Baseline | 52.3 (29.2) | 52.9 (28.8) | 52.6 (30.4) | 50.3 (28.8) | 0.943 |
| Follow-up | 26.1 (26.2) | 24.5 (23.9) | 23.8 (24.2) | 35.5 (34.4) | 0.266 |
| Leg pain, mean (SD) |  |  |  |  |  |
| Baseline | 67.2 (26.7) | 67.3 (28.7) | 72.2 (23.2) | 56.5 (26.8) | 0.073 |
| Follow-up | 27.5 (29.1) | 22.7 (28.1) | 27.2 (27.2) | 38.6 (34.2) | 0.198 |
| ODI, mean (SD) |  |  |  |  |  |
| Baseline | 45.4 (16.4) | 45.3 (17.2) | 46.8 (15.3) | 42.1 (17.1) | 0.505 |
| Follow-up | 16.1 (16.7) | 13.7 (13.7) | 16.0 (16.8) | 21.6 (21.3) | 0.235 |
| EQ-index, mean (SD) |  |  |  |  |  |
| Baseline | 0.50 (0.15) | 0.50 (0.16) | 0.49 (0.14) | 0.49 (0.15) | 0.982 |
| Follow-up | 0.73 (0.20) | 0.74 (0.21) | 0.74 (0.17) | 0.68 (0.23) | 0.537 |
| EQ-VAS, mean (SD) |  |  |  |  |  |
| Baseline | 45.8 (22.7) | 47.0 (22.4) | 43.3 (22.5) | 49.2 (24.3) | 0.518 |
| Follow-up | 72.7 (23.2) | 74.4 (19.7) | 70.9 (25.8) | 73.3 (25.2) | 0.794 |

Supplemental Table 7. Unadjusted pre- and post-operative patient-reported outcome measures (PROMs) for the total study sample and by group.

^a^ Compared using one-way ANOVA.

References

1. Zehra U, Cheung JPY, Bow C, Lu W, Samartzis D. Multidimensional vertebral endplate defects are associated with disc degeneration, modic changes, facet joint abnormalities, and pain. J Orthop Res. 2019;37:1080–9.

2. Farshad-Amacker NA, Hughes A, Herzog RJ, Seifert B, Farshad M. The intervertebral disc, the endplates and the vertebral bone marrow as a unit in the process of degeneration. Eur Radiol. 2017;27:2507–20.

3. Rajasekaran S, Venkatadass K, Naresh Babu J, Ganesh K, Shetty AP. Pharmacological enhancement of disc diffusion and differentiation of healthy, ageing and degenerated discs. Eur Spine J. 2008;17:626–43.

4. Määttä JH, Karppinen JI, Luk KDK, Cheung KMC, Samartzis D. Phenotype profiling of Modic changes of the lumbar spine and its association with other MRI phenotypes: a large-scale population-based study. Spine J. 2015;15:1933–42.

5. Modic MT, Steinberg PM, Ross JS, Masaryk TJ, Carter JR. Degenerative disk disease: assessment of changes in vertebral body marrow with MR imaging. Radiology. 1988;166:193–9.

6. Perilli E, Parkinson IH, Truong LH, Chong KC, Fazzalari NL, Osti OL. Modic (endplate) changes in the lumbar spine: bone micro-architecture and remodelling. Eur Spine J. 2015;24:1926–34.

7. Saukkonen J, Määttä J, Oura P, Kyllönen E, Tervonen O, Niinimäki J, et al. Association Between Modic Changes and Low Back Pain in Middle Age. Spine (Phila Pa 1976). 2020;45:1360–7.

8. Mera Y, Teraguchi M, Hashizume H, Oka H, Muraki S, Akune T, et al. Association between types of Modic changes in the lumbar region and low back pain in a large cohort: the Wakayama spine study. Eur Spine J. 2021;30:1011–7.

9. Pfirrmann CWA, Metzdorf A, Zanetti M, Hodler J, Boos N. Magnetic resonance classification of lumbar intervertebral disc degeneration. Spine (Phila Pa 1976). 2001;26:1873–8.

10. Jamaludin A, Kadir T, Zisserman A, McCall I, Williams FMK, Lang H, et al. ISSLS PRIZE in Clinical Science 2023: comparison of degenerative MRI features of the intervertebral disc between those with and without chronic low back pain. An exploratory study of two large female populations using automated annotation. Eur Spine J. 2023;32:1504–16.
